# Architectural ruins: geoculture of the anatomy of buildings as illustrated by Casa Ippolito, Malta

**DOI:** 10.1186/s40494-021-00500-9

**Published:** 2021-03-02

**Authors:** Lino Bianco

**Affiliations:** 1 Faculty of the Built Environment, University of Malta, Msida, 2080 Malta; 2 Faculty of Architecture, University of Architecture, Civil Engineering and Geodesy, 1 Hristo Smirnenski Blvd, Sofia, 1164 Bulgaria

**Keywords:** Ruins, Anatomy of buildings, Traditional building materials, Architectural history, Secondary geodiversity, Anthropogenic geodiversity, Geoculture, Central mediterranean, Malta, Casa Ippolito

## Abstract

Ruins are a statement on the building materials used and the construction method employed. Casa Ippolito, now in ruins, is typical of 17th-century Maltese aristocratic country residences. It represents an illustration of secondary or anthropogenic geodiversity. This paper scrutinises these ruins as a primary source in reconstructing the building’s architecture. The methodology involved on-site geographical surveying, including visual inspection and non-invasive tests, a geological survey of the local lithostratigraphy, and examination of notarial deeds and secondary sources to support findings about the building’s history as read from its ruins. An unmanned aerial vehicle was used to digitally record the parlous state of the architectural structure and karsten tubes were used to quantify the surface porosity of the limestone. The results are expressed from four perspectives. The anatomy of Casa Ippolito, as revealed in its ruins, provides a cross-section of its building history and shows two distinct phases in its construction. The tissue of Casa Ippolito—the building elements and materials—speaks of the knowledge of raw materials and their properties among the builders who worked on both phases. The architectural history of Casa Ippolito reveals how it supported its inhabitants’ wellbeing in terms of shelter, water and food. Finally, the ruins in their present state bring to the fore the site’s potential for cultural tourism. This case study aims to show that such ruins are not just geocultural remains of historical built fabric. They are open wounds in the built structure; they underpin the anatomy of the building and support insights into its former dynamics. Ruins offer an essay in material culture and building physics. Architectural ruins of masonry structures are anthropogenic discourse rendered in stone which facilitate not only the reconstruction of spaces but also places for human users; they are a statement on the wellbeing of humanity throughout history.

## Introduction

Built heritage, rendered in stone, is an anthropogenic, geocultural statement of humanity. It represents a lithological, industrial, archaeological and historical testimony to sustainable architectural science. The present article deals with the geocultural aspects of stone buildings, that is, those that are primarily composed of geological materials drawn directly from nature, such as dimension stones hewn from igneous, metamorphic and sedimentary strata, as well as processed geoproducts such as clay bricks and tiles or lime mortar.

The architectural evolution of Malta is based on the use of local limestone. For millennia, the lithostratigraphic formation outcrops have been exploited for construction and in the manufacture of internal household features such as stone shelves and stoves [1]. Long before geology was recognised as an academic discipline, the builders of Malta held sufficient knowledge of the properties of the local limestone to distinguish between the various geological strata. Even the megalithic builders of Malta differentiated between the outcropping lithostratigraphic formations, using the softer, easily hewn Lower Globigerina Limestone (LGL) for decorated interiors and the harder Coralline Limestone on the less elaborate exteriors. Over the millenia, a corpus of oral knowledge based on empirical observations gradually developed. Builders learned to distinguish between the various lithostratigraphic strata and the diverse beds within them on the basis of the physical properties of the stones such as strength and durability. The latter aspect was considered significant not only in the building of military and public edifices but also in civil structures, most notably the residences of wealthy and aristocratic citizenry.

**Fig. 1:**
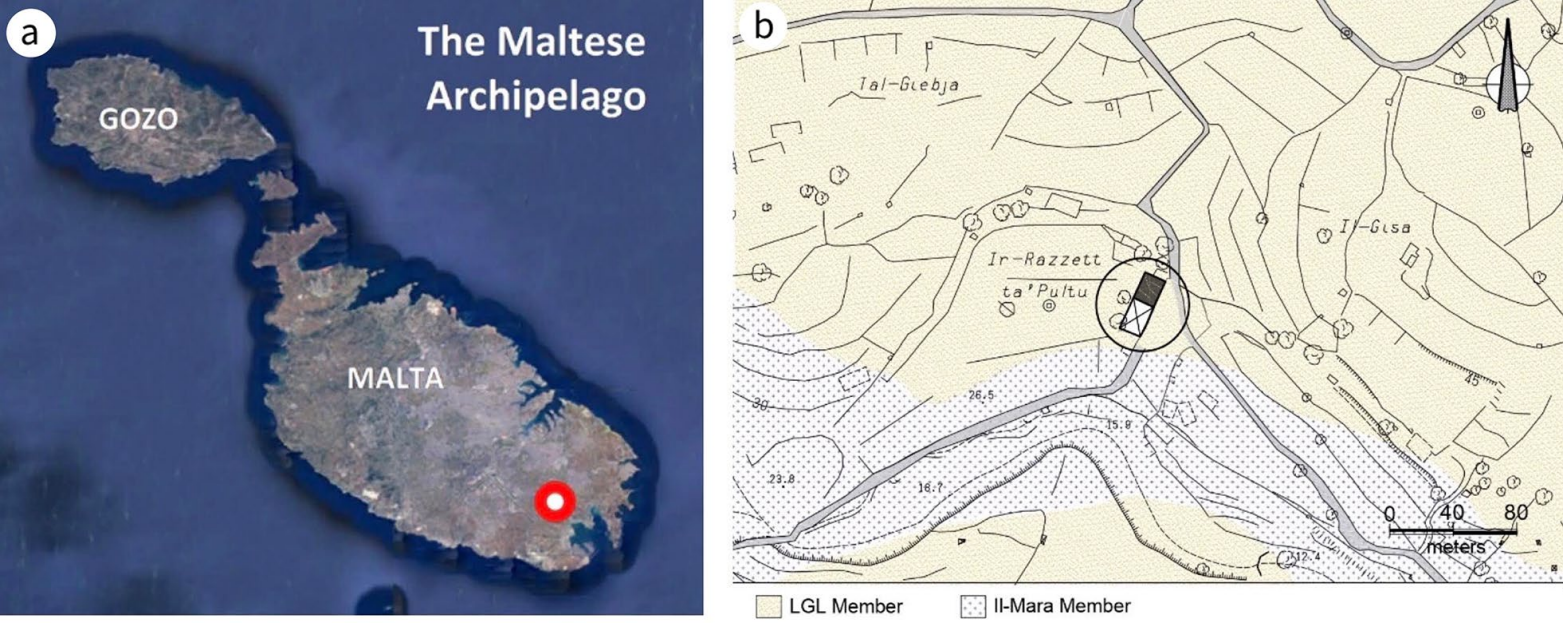
**a** Location of site marked in red (^©^Google Earth). **b** Site location map: Casa Ippolito is circled (based on a geological map of the Maltese Islands [22]

By focusing on the ruins of seventeenth-century residential architecture in Malta, this paper appraises various elements in building construction exposed in the remains of Casa Ippolito (Fig. 1), the theme of a recent publication [2]. Mario Buhagiar, an academic versed in the history of art and architecture in Malta, considers this building the “most interesting example of seventeenth-century Maltese rural architecture” ([3], 260). Generally, when a building is erected, its structure veils certain parts, rendering them invisible. Consequently, a partly demolished wall not only offers insight into the causes and process of its collapse: it provides a cross-section of the construction history of the building. It is, effectively, an essay in building engineering and construction details, delineating the history of architecture through building materials, both local and imported, and how they were brought together to form the anatomy of the building. The ruins of Casa Ippolito are approached from the perspective of geoculture and anthropogenic geodiversity—that is, how the building relates to its physical surroundings and how humans have exploited and adapted to the geological and climatic conditions of the location. To this end, these ruins and their context are evaluated through non-invasive tests and field surveys, the latter supplemented by unmanned aerial vehicle (UAV) or drone—a method increasingly used in geomorphology to access, assess, manage and research sites located in difficult terrain [4].

In the literature on the history of architecture, the significance of primary sources, notably archival material, is widely emphasised. Less attention is generally paid to the physical context—the building itself and its immediate environs—even though these represent primary sources of crucial importance. This applies as much to architectural ruins as to complete buildings. If architecture is an essay in the anatomy of a building, then the act of dismantling a structure is open surgery, revealing the mechanics of architecture, and architectural ruins are open wounds.

More recently, the concept of geodiversity has been deployed to explore the interrelations between geology and built heritage. Murray Gray defines geodiversity as “the natural range (diversity) of geological (rocks, minerals, fossils), geomorphological (landform, topography, physical processes), soil and hydrological features. It includes their assemblages, structures, systems and contributions to landscapes” ([5]: 12). Kubalíková and Kirchner note that geodiversity is a significant asset for geotouristic and geoeducational interventions [6]; as Gray points out, it is “an integral part of natural capital” ([7]: 669), a point reiterated by Brilha et al. [8]. Of particular relevance to this study is Gray’s division of the concept of geodiversity into natural, or primary geodiversity—that is, natural features without anthropogenic interference—and secondary (manmade or anthropogenic) geodiversity [9]. Secondary geodiversity may be defined as “the range/diversity of the manmade/anthropogenic landforms, including their assemblages, relationships, structures and systems” ([10]: 64). Buildings or other anthropogenic structures built directly from raw materials such as limestone represent part of an area’s secondary or anthropogenic geodiversity.

Following independence in 1964, the dominant form of tourism in Malta was “sun and sea”. In the early 1990s, in response to competition from newer destinations, the Government of Malta took a more diverse approach, shifting the focus towards cultural and heritage tourism [11]. The Malta Tourism Authority (MTA) was set up in 1999, and in 2002 the process of ‘repositioning’ the island as a cultural heritage destination was initiated [12]. A four-point programme was launched to upgrade principal heritage sites, aimed specifically at improving interpretation, accessibility, conservation and promotion. Protective shelters were erected over the megalithic complexes at Ħaġar Qim, Mnajdra and Tarxien, and visitor centres were built to improve site interpretation at Ħaġar Qim [13] and Ġgantija. Significant investment went into Valletta, especially from 2012 after it was designated European Capital of Culture 2018.

Malta’s tourism policy defines culture as a primary asset for tourism. It treats heritage and culture as features which attract tourists, rather than as add-ons to beach resort holidays, advocating a “focus on our cultural offering as it contributes to attract tourists who are interested in heritage, in our local traditions, in contemporary art and creativity and in all those spheres that create cultural distinctiveness for the Maltese islands” [14]. However, the desire to move away from a beach resort tourism offer to one based on heritage and culture is only mildly reflected in the data on tourists’ motivations [15]. Despite an increase in absolute numbers, the proportion of culture and heritage tourists over the period 2007 and 2011 has remained stable, at around 15% of all holiday visits to Malta [16]. Table 1 presents results of MTA’s traveller survey, which examined the motivations of tourists visiting Malta. The data show that from 2016 to 2019 there was only an incremental increase in the proportion of travellers motivated by cultural tourism, reinforcing that this remains a niche activity in Malta. In this context, what role can ruins such as Casa Ippolito play in Malta’s cultural tourism offer? This query underlines the importance of understanding secondary geodiversity elements and the geotouristic value of the ruins.

**Table 1:** Tourists travelling for (i) culture and (ii) sun and culture

	2016		2017		2018		2019	
	Number	%	Number	%	Number	%	Number	%
Culture only	169,121	8.6	204,317	9.0	263,535	10.1	297,693	10.8
Sun and culture	943,093	48.0	1,127,396	49.6	1,304,994	50.2	1,356,365	49.3
Total	1,965,928	100.0	2,273,838	100.0	2,598,690	100.0	2,753,240	100.0

Source: E-mail, dated 4 December 2020, from Tania Sultana (Head of Research, Research Unit, Malta Tourism Authority) to author

### Study area

Located 100 km south of Sicily and 300 km north of Libya, Malta is a Central Mediterranean island rich in cultural and natural heritage and the main island of the Maltese archipelago (Fig. 1a). Malta’s semi-arid climate is characteristically Mediterranean, with mild, humid winters and hot, dry summers. Annual rainfall is ~ 400–700 mm and the prevailing wind direction in winter is north-westerly (40%), with speeds typically up to 20 knots (37 kph) [17–19].

Due to its geographical position, Malta has historically been at the crossroads of the various Mediterranean civilisations, acting as a trading post between the northern and the southern shores. Its built heritage reflects these diverse cultures, with the oldest sites dating from the Neolithic Age. Malta is the seat of the oldest known free-standing architectural buildings (3600–2500 BC), predating the pyramids of Egypt (2700–1700 BC) by a millennium. Some of these sites are listed as UNESCO World Heritage sites, including the megalithic temples of Ħaġar Qim, Mnajdra and Tarxien, on the island of Malta, and Ġgantija on Gozo [20]. More recent Maltese sites listed by UNESCO include the island’s Late Renaissance capital, Valletta, erected by the Hospitaller Order of St. John (1530–1798), and considered one of the most concentrated historical areas in the world [21]. The period when Malta was ruled by the Order is generally recognised as being the richest phase in terms of art and culture, including architecture.

Malta’s geology is of shallow marine Oligo-Miocene sedimentary origin [23]. The main lithostratigraphical subdivisions and their physical properties are given in Table 2 [22, 24]. There are three limestone formations, namely Upper Coralline, Globigerina and Lower Coralline, all of which have diverse physical, textural, chemical and mineralogical characteristics and a number of separate members. The use of Upper Coralline in 17th- and 18th-century Malta has been addressed by Bianco [25]. The latter two formations are both used in Casa Ippolito. The Casa is built on the LGL (Miocene, Aquitanian) which overlies the Il-Mara Member (MM) (Oligocene, Chattian), the upper member of the Lower Coralline Limestone (LCL) (Fig. 1b). West of the site, along the flank of Wied Dalam wadi, there are signs of industrial archaeology. Traces of old open-pit mining operations can be seen and the limestone used in nineteenth-century country houses in the vicinity has been attributed to quarries in this area [26].

**Table 2:** Lithostratigraphical subdivisions of the geology of Malta [22] and respective physical properties ([24]: 165)

Formation	Porosity (%)	Compressive uniaxial strength (N/mm^2^) (dry)	Flexural strength (N/mm^2^) (dry)	Member	Age
Upper coralline limestone	2.4–32.3	8.8–67.2	NA	Ġebel Imbark	Messinian
				Tal-Pitkal	Tortonian/Messinian
				Mtarfa	Tortonian
				Għajn Melel	Tortonian
Greensand	23.8–32.4	NA	NA		Tortonian
Blue clay	X	X	X		Langhian/Tortonian
Globigerina Limestone	26.2–37.4	8.9–22.0	1.1–4.7	Upper Globigerina	Burdigalian/Langhian
				Middle Globigerina	Aquitanian/Burdigalia
				Lower Globigerina	Aquitanian
Lower coralline limestone	1.8–28.3	6.8–105.0	NA	Il-Mara	Chattian
				Xlendi	Chattian
				Attard	Chattian
				Magħlaq	Chattian

*X* not applicable, *NA* not available

Beds of LGL and MM occur in the immediate vicinity, south of the site, and their diagnostic properties and uses in the building industry are given in Table 3. LGL is pale cream to yellow in colour and composed of planktonic foraminiferal packstones, rapidly becoming wackestones above the base. MM is composed of tabular beds of pale cream to pale grey carbonate mudstones, wackestones and packstones. LGL is characterized by calcareous plankton [27]. The dominant mineralogy is calcite with minor inclusions of quartz, feldspar, muscovite, kaolinite, illite, smectite and glauconite. The percentage of non-carbonate content increases with decreasing quality [28]. LCL, with calcite as the bulk mineral, is more compact and less porous than LGL, one of the soft building limestones found in the Mediterranean Basin. Collectively known as ‘franka’ (translated as ‘freestone’), its outcrops occur over a significant part of the island. Whilst franka is used as a generic term for LGL, inferior-quality lithostratigraphical beds known as ‘sol’ (also written as ‘soll’) occur within this member, presenting regularly at circa 12 m intervals. Sol is more absorbent than the higher quality franka [29] and comes in two types: ‘sol aħmar’ (red sol) and ‘sol ikħal’ (blue sol) [30, 31]. Both are less weather resistant than the best quality franka and while sol aħmar can be used as a dimension stone in foundations and at building levels over 1.2 m above the ground, sol ikħal does not withstand exposure to the elements. The first comprehensive study of the petrographical, mineralogical, geochemical and physical characteristics of LGL was undertaken at the University of Leicester [28]. The resultant material characteristics, together with a geohistorical retrospective analysis, are useful in identifying the provenance of LGL in heritage buildings [32, 33].

**Table 3:** Beds of LGL and LCL present on the Casa Ippolito site and its surrounding environs [1, 34]

Formation	Bed	Characteristics	Uses
Lower globigerina limestone	9_2_	Dark stone; does not withstand exposure	Foundations and other instances where protection from the atmosphere is present
	9_1_	Pale yellow limestone, turns into light reddish-brown colour after some time; composed of minute fossils; easily split into thin slabs; hardens when exposed to air; weathers very well; no fossils are present except for remains of saurians etc. and a few shells	Building stone, paving stone, masonry lintels, and roofing slabs to span between masonry arches and beams
Lower coralline limestone	1	Transition (Scutella) bed; soft; often mixed and merging into the calcareous sands of the overlying stratum; fine-grained; not durable; Echini project outwards when stone decays away	Not much used in building

## Methodology

### Case study

Casa Ippolito was an aristocratic rural residence erected in ashlar masonry [2]. Historically, this mode of construction was used in monumental architecture and in higher-quality residential buildings [35]. It was the residence of Ippolito Novantieri, a wealthy aristocrat from Syracuse [3]. The house was probably located in the artistic garden known at the time as “il Ġnien ta Ċiakra”.^1^ The original inscription above the building’s main entrance bore the date 1664, most likely giving the year the construction works were completed. A publication on old towers of Malta issued a century ago included Casa Ippolito as a fortified house [36]. This classification was reiterated nearly half a century later in the Protective Inventory of the European Cultural Heritage: “A fortified country house with a basement and two floors, consisting of a large courtyard, a mill, stables, a cow sty divided in two parts, four rooms and a kitchen”.^2^ From the provenience listed in the title, dated to a century ago, there is no mention that any portion of the land forming part of the same property was ever sold or transferred to third parties.^3^

**Fig. 2:**
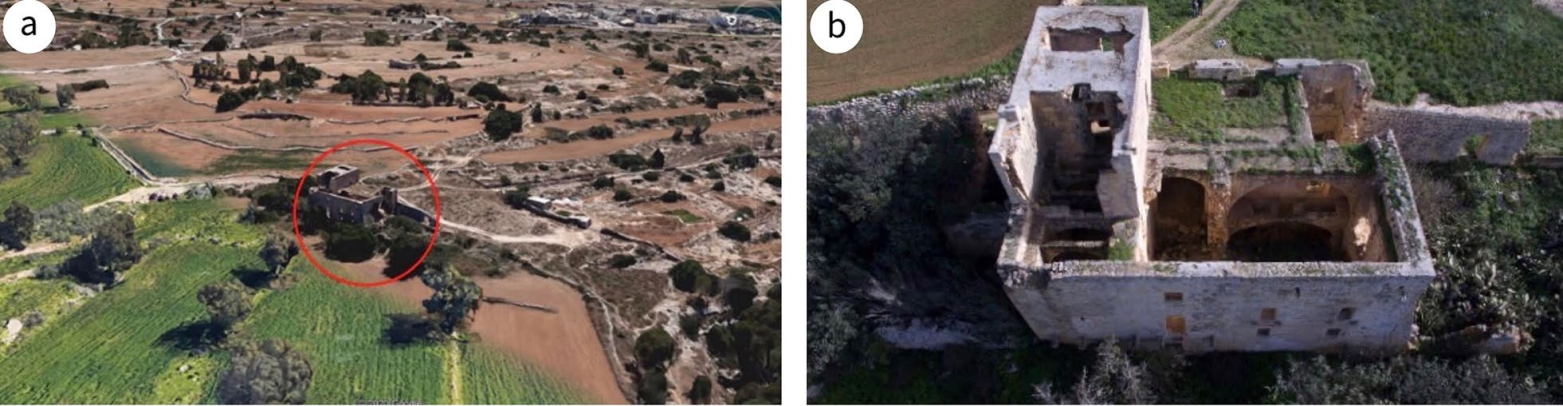
**a** Aerial perspective of the site (circled in red) including the surrounding environs (^©^Google Earth). **b** drone view of the site from the north-west (^©^Joe Fenech)

The site is located on the flank of Żembaq Valley, west of the Roman ruins at Ta’ Kaccatura, on the limits of Birżebbuġa (Fig. 2a). By the time it fell into disuse, around 1919, the inscription on the main doorway had already deteriorated, the text “almost entirely illegible due to atmospheric erosion”.^4^ Since then, the building has been abandoned and is now reduced to a ruin, with most of the roofs, some floors and various walls having collapsed, while others are in a dangerous state (Fig. 2b). By 1967, the main fabric, subsidiary portions, roof and the interior were already in a bad state of preservation^5^ and vandalism had added its toll. In 1998, it was scheduled as a Grade 1 building [37], awarding it the highest level of protection in Maltese legislation, listed among buildings of “outstanding historical or architectural interest that shall be preserved in their entirety. Demolition or alterations which impair the setting or change the external or internal appearance, including anything contained within the curtilage of the building, will not be allowed. Any interventions allowed must be directed to their scientific restoration and rehabilitation. Internal structural alterations will only be allowed in exceptional circumstances where this is paramount for reasons of keeping the building in active use” ([38]: 88).

**Fig. 3:**
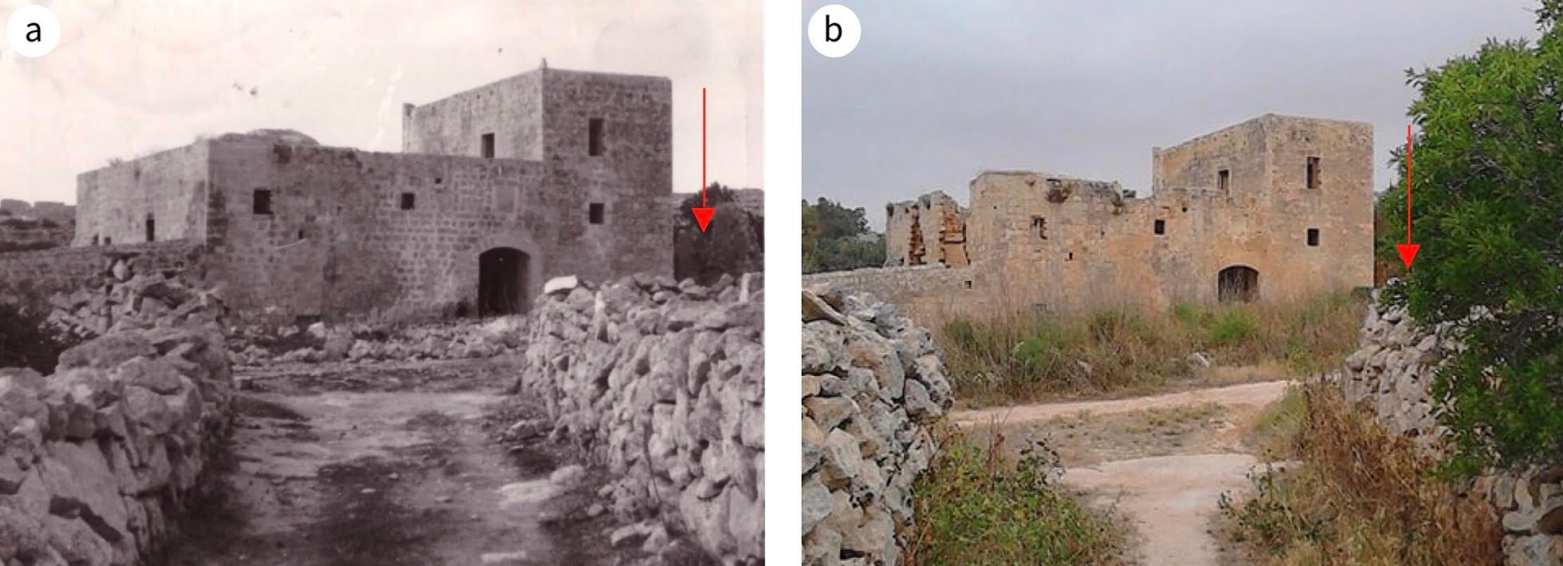
South-east facing elevation **a** in 1967 (NMA: Facade to East.) (^©^Heritage Malta), and **b** at present. Position of ruin of the ‘remissa’ is indicated by marker

**Fig. 4:**
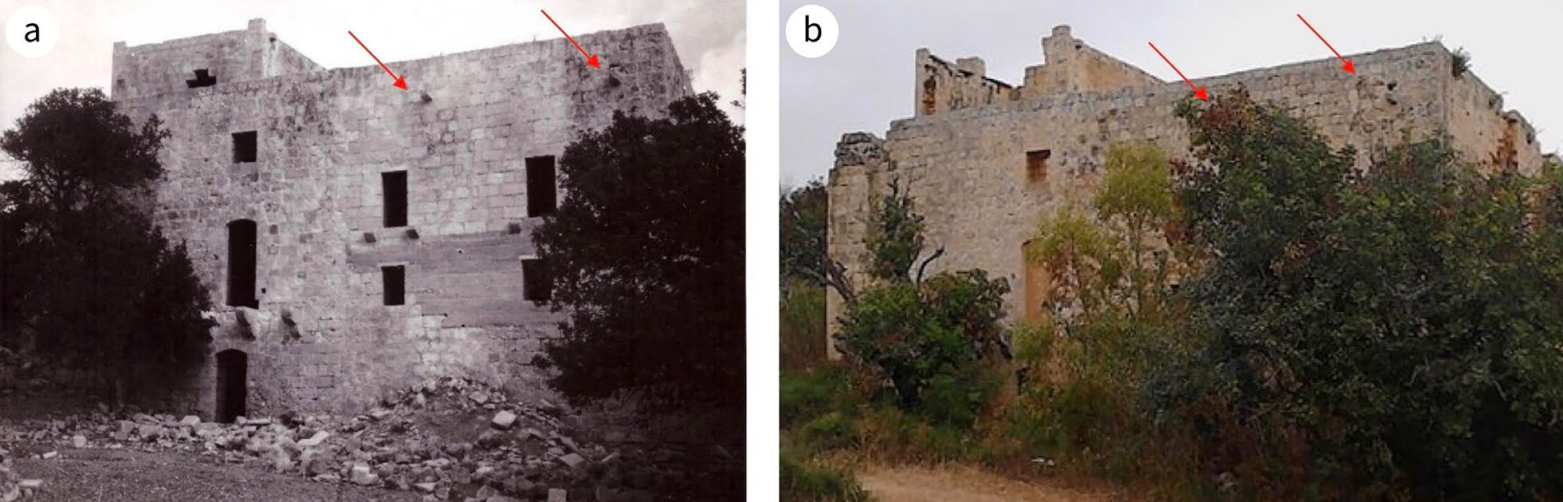
North-west elevation **a** in 1967 (NMA: Facade to West.) (^©^Heritage Malta), and b at present. This elevation is exposed to the direction of the prevailing wind; masonry water spouts are indicated by markers

Architecturally, the building is rectangular in plan; on its south-west boundary there is a yard with a separate access from the public country road. The path from the access ran through to the opposite side of the yard and led to the surrounding fields that form part of the property. The yard is set at a lower level, following the natural gradient of the site topology, thus preventing rainwater and soil dampness percolating to the upper levels. The south-east elevation of the building runs alongside the public road (Fig. 3) while the opposite side, facing north-west, overlooks agrarian land (Fig. 4). Adjacent to the house, along its north-east elevation and to the right of the entrance, there is a ruin, and adjacent to the south-west facing wall of the yard, at the corner with the road, there was an underground cistern. Now partly collapsed, it was hewed manually and roofed with masonry slabs supported by masonry arches, or to use anatomical terminology, ribs.

**Fig. 5:**
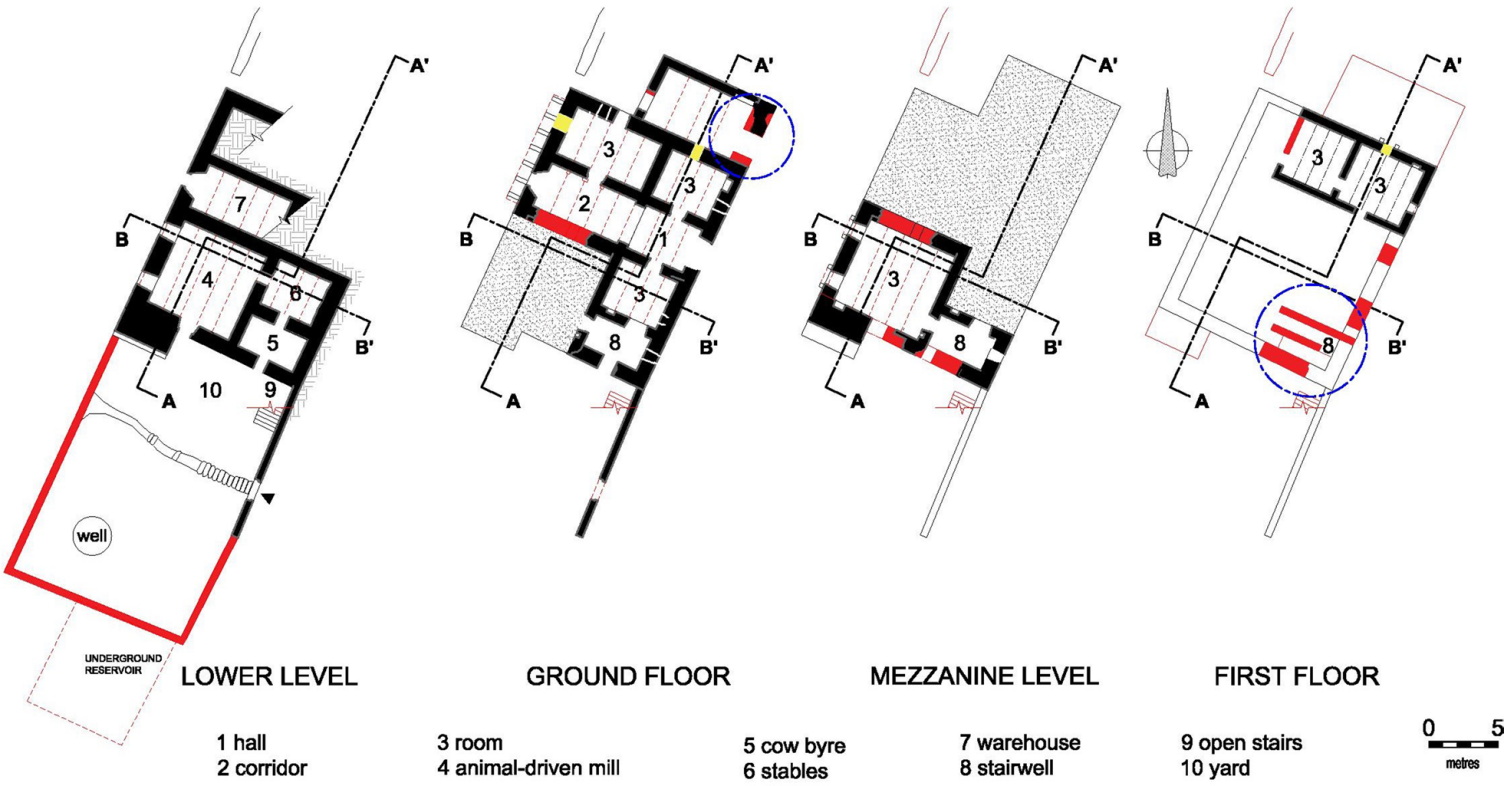
Plans; approximate layout is indicated by the circle

**Fig. 6:**
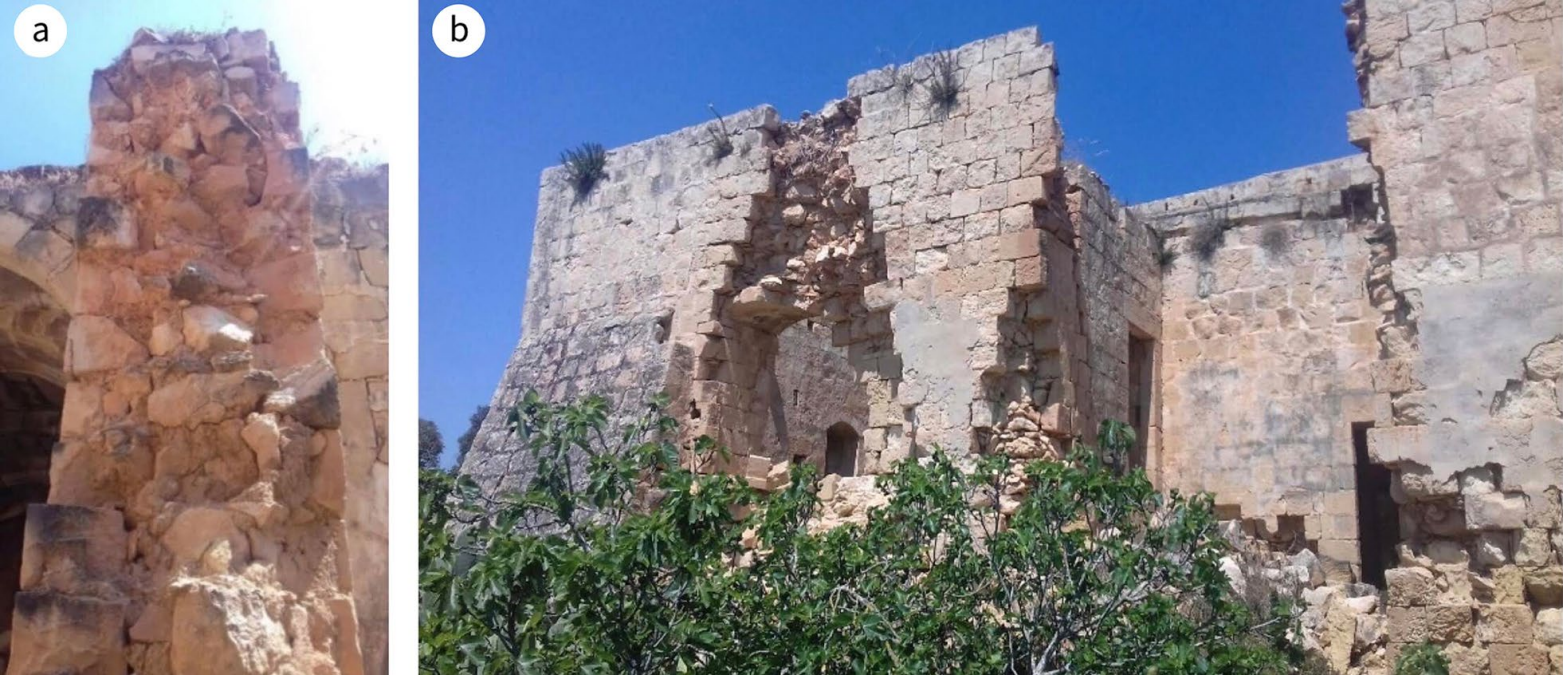
**a** Section through the collapsed internal wall between the corridor and the mill (and the overlying mezzanine). **b** Buttress-like structure along part of the south-west facing elevation

**Fig. 7:**
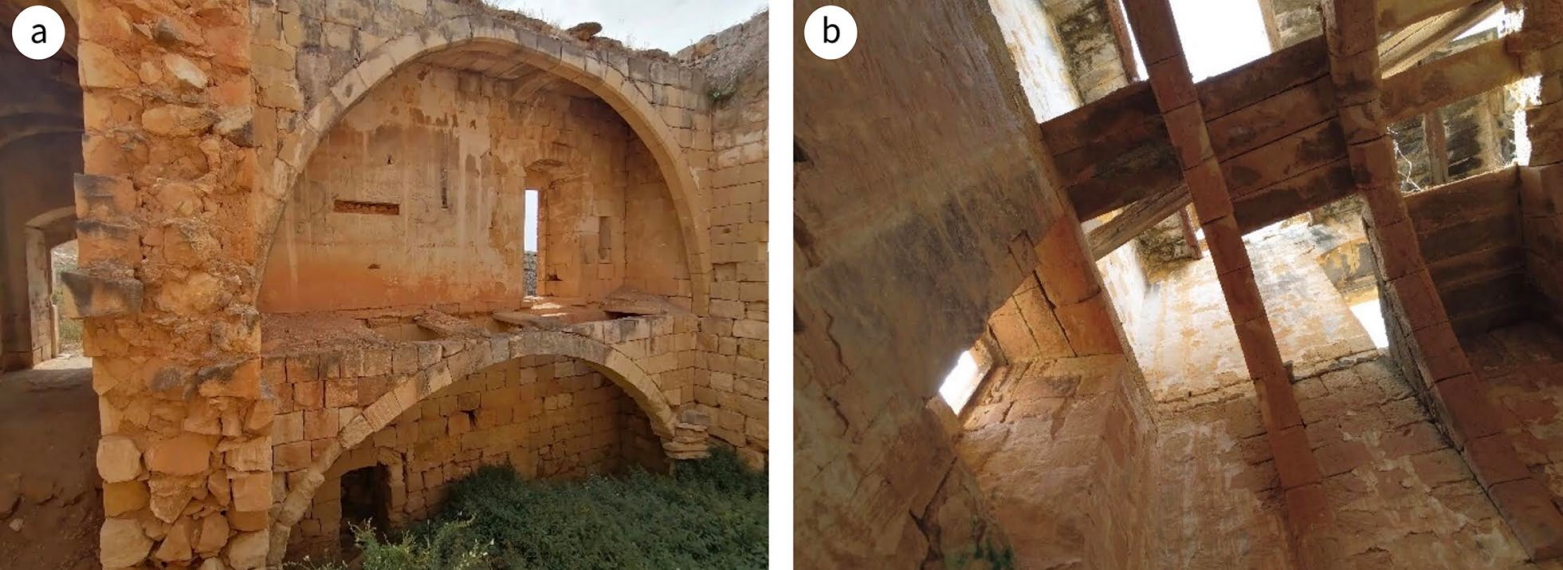
Collapsed roofs of **a** the mill room and overlying space, and **b** the first room on the right on entering the building; note the timber beams across the uppermost roof (^©^Alessandra Bianco)

The residence was a load-bearing masonry structure erected in a traditional building style dating from mediaeval Malta. It was constructed on four levels: lower floor, ground-level, mezzanine and second storey (or first floor), the lower floor being accessible from the yard (Fig. 5). The entrance led to a hall and a corridor ending with an open balcony overlooking the agrarian land. The second room on the right upon entering the hall was used as a kitchen/dining room, indicated by a built-in chimney embedded in the wall and running up to the roof of the first level. The walls are thick (Fig. 6a) and on the south-west side there is a buttress-like structure (Fig. 6b), which suggests the premises might have been fortified, justifying Mifsud’s inclusion of it in his publication [36]. The lower, ground and mezzanine levels were roofed over by masonry slabs (Maltese: xorok; singular: xriek), now mostly collapsed, supported by semi-circular masonry arches (Fig. 7a). The second storey was roofed in a similar manner but supported instead by timber beams (Fig. 7b). The first levels, up to the roof of the ground floor, were linked by an internal staircase, while the first floor was accessed from that roof. The access and rooms of the second storey are typical of dry-rooms used for the storage of fodder (Maltese: għorof; singular: għorfa). This explains why the roof overlying the ground floor had a parapet wall around it, making it into a terrace, while the roof of the għorof has no such structure, being simply a roof laid with falls in a west-facing direction. This orientation could be inferred from the masonry drain element which directs the rain water from the roof of the għorof to that of the ground-level floor via a clay pipe. It is then drained via masonry water spouts located along the north-western elevation (Fig. 4). An uncovered flight of stairs ran from the stairwell to the yard.

### Fieldwork and desk studies

In terms of fieldwork, a visual survey and various non-invasive tests were carried out on site. To comprehend the building and its physical environment, a detailed evaluation of the ruins was carried out and the extant site mapped out. Given the safety hazards presented by the parlous state of the ruins, the survey was supplemented by photographs taken from the ground and from a UAV.

The geological and pedological features of the immediate environs were assessed, complemented by data from the latest geological map [22] and an old but highly accurate soil map [39]. Three simple but effective methods were applied to assess the types of limestone present: (i) karsten tubes were used to quantify water permeability, and hence the surface porosity of the limestone, at 29 different locations; (ii) geological hand lenses (10 × folding pocket magnifiers) were used to visually examine 16 dimension stones and the respective host fabrics; and (iii) a geological hammer was used to break up rocks from the surrounding landscape but was not used on the building’s fabric itself. Munsell Soil Colour Charts were used to identify the soils in the area and the composition of the infill—where exposed—in the double-leafed walls. In total, 6 samples from the infill and 7 samples from the surrounding fields were assessed.

In terms of desk research, the following documents were consulted:

1. Ordinance Survey sheet 5666, 1973 and 1988 versions, both at a scale of 1:2,500.
2. Official aerial photographs and orthophotos available at the Mapping Unit of the Planning Authority, Malta, the latter available online at http://geoserver.pa.org.mt/publicgeoserver.
3. Various documents, including dated photographs, from the case file on Casa Ippolito, accessible at the reserve collection of the National Museum of Archaeology (NMA), Heritage Malta, Valletta.

To plot the progressive collapse of the roofs, aerial photographs and, where available, orthophotos were used. Although the latter were derived from the former, orthophotos are more accurate than unprocessed photographs due to distortions arising from the aerial survey. No archival sources on the building were obtainable other than a number of notarial deeds, the earliest and latest dated 1726 and 1899, respectively; however secondary scholarly sources were drawn upon to aid in the interpretation of the findings, notably Hughes [40], Hoppen [41] and Mahoney [42].

## Results and discussion

The results and discussion were grouped under three themes:

1. The anatomy of the building.
2. The tissue of the architecture.
3. Geoculture as wellbeing.

This is followed by a discussion on architectural ruins as a cultural tourism product, reflecting the priorities and trends of the tourism market in Malta, a destination which has, since the turn of the millennium, been seeking to offer something more diverse than a mere sun and sea destination.

### The anatomy of the building

A public deed dated 1893, when the residence was still fit for habitation, states that the house and surrounding lands had a superficial area of 36 tumoli, 4 mondelli and 2 misure: equivalent to just over 41,000 m^2^. The same document includes the following description:

“the space occupied by the house consists of fourteen fields with walls, a cistern, and a house containing a large courtyard with two doors, one facing the road and the other on the ground (Italian: terreno), a cow byre (Italian: bovile) divided into two, one uncovered staircase leading to a room on part of the said byre, a horse mill, two stables, a flight of uncovered stairs leading to the floor at road level, which becomes the ground floor, and a warehouse that has ingress from the said ground.

“The ground floor, which is above the aforementioned amenities, contains an entrance with a door onto the street—two side bedrooms, a kitchen, a staircase leading to a room above the horse mill, and a continuation of the staircase to the terraces, and from these you go to two rooms, and to an open loggia, overlying the ground floor, plus a ‘remissa’ with a door to the street, and a stable with entrance from the fields”.^6^

The description of the ‘remissa’—a permanent roofed-over space used as a store and a garage for carts—fits the present ruin adjacent to the house, although such a structure is not shown on the site plan attached to the said deed. This implies that, up to 1893, this structure was still in a good state of repair.

The survey of Casa Ippolito established the main configuration of the building. Two queries emerged: when was the remissa erected, and what was the extent of the collapsed boundary walls of the yard? In the ruins of the remissa, one can still read the spring of the arches from the wall of the house, but was this space erected prior to, simultaneously with or after the dwelling? Factual observations indicate that it was constructed post-1664. There was a well-formed window in the wall which overlooked the site of the ruin and that was blocked prior to roofing the remissa. It was not a dummy aperture; it was realised in fine ashlar on the exterior and unfinished on the interior. The current remains of the walls of the yard coincide with the plot on the 1988 Ordinance Survey sheet. The sheet issued in 1973 shows the wall of the yard parallel to the public country road extending further south, but there were no traces to show whether or not this ran the whole length of the yard or how it joined the wall running along the road. The site plan attached to the deed of 1893 confirmed that this was the length at the time and that it was joined through a straight line.^7^

**Fig. 8:**
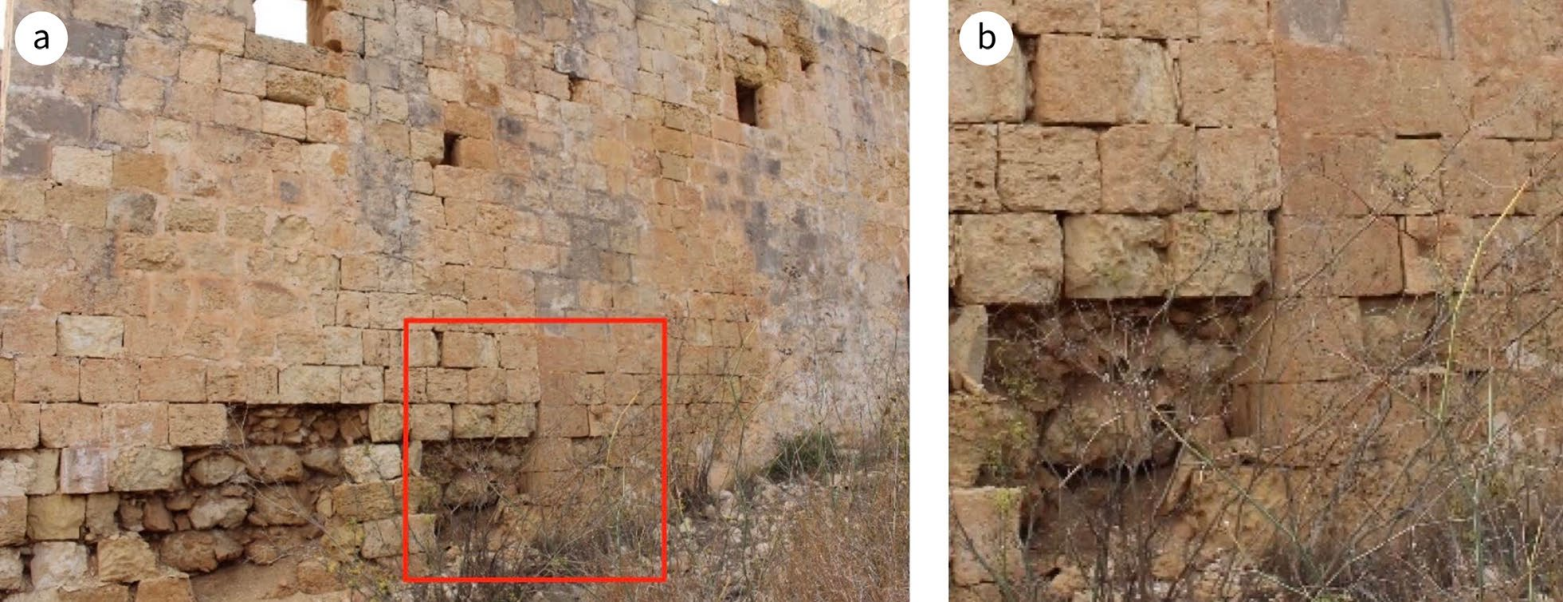
**a** Absence of proper mechanical bonding between dimension stones along the exterior denoting a change in phase in the building construction, **b** detail of **a**

A visual inspection of Casa Ippolito implied there were two construction phases in its erection. The later phase included the stairwell, the mill and the mezzanine-level room that overlies it. This phase is recognisable by:

1. an absence of bond stones both on the exterior (Fig. 8) and on the interior up to the level of the lintel of the door of the room on the left upon entering the main entrance;
2. a change in the quality of the LGL used, differentiated by its weathering characteristics; and
3. the fact that the internal wall common with the mill and the overlying room is the same thickness as the external walls, implying that it was originally an external wall. The internal walls elsewhere in the house are narrower.

The present state of the ruin was established from drone images (for example, Fig. 2b). The period over which the collapse took place and the percentage area of the total roof are included in Table 4. Given their weak resistance to impact, when the xorok collapsed they caused the subsequent collapse of the underlying floors. The earliest collapse took place between the years 1967 and 1978 and the latest between 2008 and 2012.

**Table 4:** Gradual collapse of Casa Ippolito (denoted in hatched colour)

Year	Scale of photos	Demolished roof/s	% of total roof
1967	1:4000		00
1978	1:10,000		05
1988	1:6000		11
1994	1:4000		16
1998^a^	1:10,000		16
2004^a^	1:15,000		20
2008^a^	1:4000		52
2012^b^	Not available		56
2016^a^	1:10,000		56

^a^Orthophotos were consulted

^b^Only orthophoto exists

All the roofs were flat, as is typical of the coastal regions of the southern Mediterranean. While precipitation, especially the absence of snow, had a bearing on the use of this type of construction, there seems to be a strong cultural element involved as well. The lack of available resources may also have contributed to the preference for flat roofs over the low-pitched roofs which characterise the northern shores of the Mediterranean.

The date of the initial collapse of Casa Ippolito is corroborated by archival photos found at the NMA, dated 1967, in which the roofs had not yet collapsed. A black-and-white image of the entrance hall included in an article by Buhagiar [3] indicates that (i) the internal wall of the room at the mezzanine level had an opening onto the corridor at ground level; and (ii) the roof of the warehouse underlying the corridor had already collapsed. A colour photo of the same view showed this internal wall stained in a green typical of algae or moss. This strongly indicates a continuous ingress of rain water over a number of years, as algae and moss require a damp environment. Rain percolation caused the double wall—which is characterised by the absence of bond stones between the two leafs and designed to take the side thrust of the masonry ribs of the mill and the overlying room—to fail by bursting outwards.

### The tissue of the architecture

All walls of Casa Ippolito were composed of two leafs of ashlar masonry. The average thickness of the external walls was 1.2 m. Stone off-cuts and other chippings, together with soil, were used as infill. Historically, the cavity wall was introduced in an attempt to solve the problem of external walls being perpetually wet, producing dampness which eventually reached the inner face. However, the local situation is very different. Rainfall is generally seasonal, with long dry periods which allow the porous local stone to quickly become bone dry. Even during the rainy season, the pattern is high intensity precipitation during short periods of stormy weather followed by long dry periods, providing ample time for the stone to dry.

**Fig. 9:**
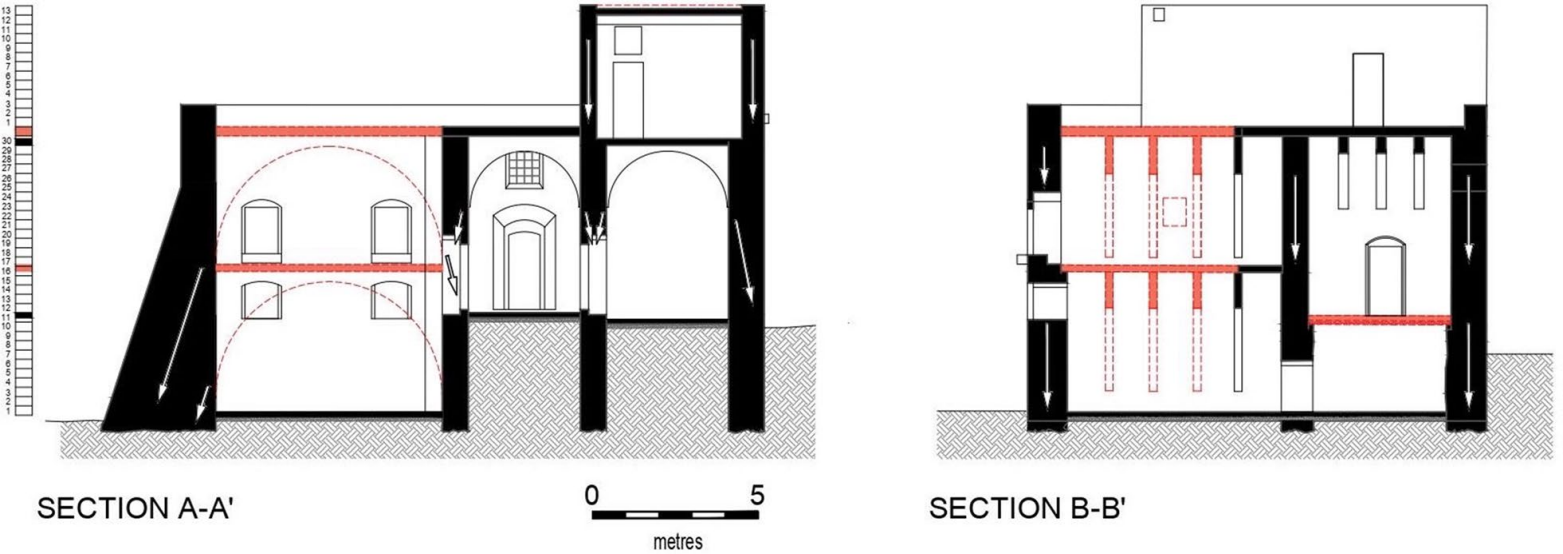
Section: the path of transfer of compressive stresses generated by the roofs is indicated by the direction of the arrows; for position of sections, see Fig. 5

Characteristic of the geologic substratum, two soil types occur in the immediate vicinity of the Casa: Xaghra Series and L-Inglin Complex, overlying the MM and the LGL, respectively [43]. Using the *Munsell Soil Colour Charts*, it was found that both types were used in the construction of Casa Ippolito’s double walls, but the presence of the former—a semi-natural reddish brown clay soil which is distinct from the L-Inglin Complex, which is anthropogenic [39]—is more frequent in the exposed sections. Unlike other heritage buildings, the internal walls were not single-leafed. The external walls were designed to satisfy a significant structural engineering consideration: to accommodate the thrust of the masonry aches (Fig. 9). The thickness of the internal walls is 0.8 m. Given that the għorof were roofed by horizontal timber beams which did not generate side thrust, the width of their external walls was less than those which form the room beneath. The buttress was not a military design, but was constructed to take the side thrust generated by the arches of the mill and the overlying room.

**Fig. 10:**
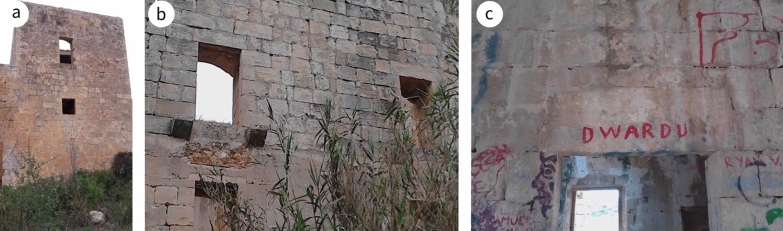
Masonry lintels: **a** the lintel could be loaded without failure in tension up to a span ≤ 0.9 m; otherwise a relieving arch was introduced, **b** the stone above the lintel was notched, **c** depth of lintel increased by 50% the height of a building course

Spanning openings such as apertures and roofs is historically one of the biggest challenges in architecture. The spanning solutions for apertures in Malta’s traditional architecture are listed in Table 5. LGL can withstand compression but is weak in tension [24]; the rule of thumb among local builders using this material was that a stone lintel could be loaded without failure in tension up to a 0.9 m maximum span; any longer than this and a relieving arch would be necessary (Salvatore Bondin, personal communication) (Fig. 10a). When such an arch was absent, “the stones directly above the lintel were often notched out so that they did not rest on the top corners of the lintel” [40: 198] (Fig. 10b). Another solution used at Casa Ippolito was to increase the depth of the lintel by 50% for a 0.9 m span [2] (Fig. 10c). Failures in masonry lintels for spans less than 0.9 m occurred due to corrosion and the subsequent expansion of the iron grills, which causes typical cracking in stone masonry—lintels, jambs, etc.—when the inserted metal corrodes. For larger openings, masonry arches were used.

**Table 5:** Spanning solutions for apertures in traditional architecture in Malta [2, 40, 42]

Span (s)	Spanning solutions
s ≤ 0.9 m	Masonry lintel
0.9 m < s ≤ 1.1 m	Relieving arch Notching of dimension stone exactly above lintel Depth of lintel increased to 1.5 times the depth of a masonry lintel for a 0.9 m span
s > 1.1 m	Masonry arch

The masonry roofing slabs were LGL dimension stones, 75 mm thick on average. These were used to traverse between masonry arches or between timber beams (Fig. 7b). Masonry ribs were stronger, not vulnerable to biological rot and more fire-resistant than wood. The length of a xriek is the crucial factor in this type of construction. The typical length in Maltese residential properties erected prior to the Second World War was around 700 mm. The strength of the slabs in tension was minimal and their stability against failure due to excessive moments was marginal, although point loads were the crucial factor. The moment generated by a point load or a distributed load on a slab 700 mm wide was minimal. In general, only in the first case would failure occur. Impact loads could be very problematic. Only the overlying layer of about 150 mm of fill, locally known as ‘torba’ and intended to spread the load, made the construction viable. Longer slabs were available but only used where the upper floor was inaccessible. Their factor of safety against failure was extremely low. The length of a xriek varied between 0.7 m and 2.0 m. A xriek of the maximum size was known as xriek tal-qasba, which translates as a cane-length roofing slab, where one cane was equivalent to 2.1 m. In cases where xorok tal-qasba were utilised, a crossbeam was often introduced as a secondary support; this was especially useful should a xriek fail. The xorok were bevelled along their length and, once placed on the ribs or beams, formed v-shaped grooves where they met. They were wedged in on all sides and the grooves were filled with a mix of lime, LGL powder and fine wet stone chippings. A layer of torba stone chippings and LGL flagstones was subsequently placed on top to uniformly distribute the load on the otherwise weak-in-tension slabs [40] since no alternative material was locally available. The use of LGL slabs as flooring material was problematic. The stone was very soft, resulting in uneven wearing of the surface; moreover, it was very porous, so dirt penetrated the surface and was almost impossible to remove.

**Fig. 11:**
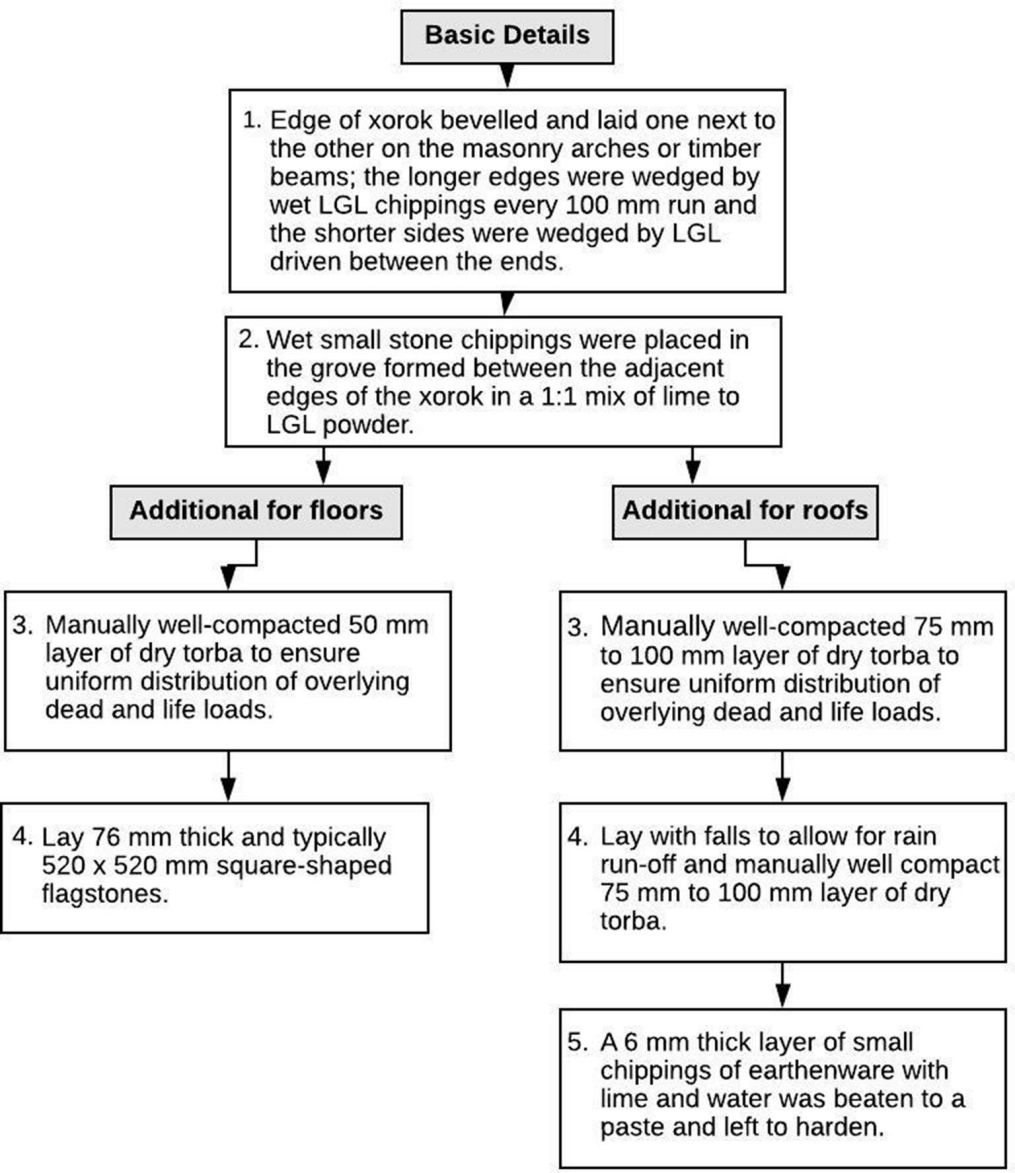
Floor and roof construction details in traditional architecture in Malta

Roofs exposed to the elements were constructed in a similar manner but were finished with a hardened paste of a hydraulic mortar mix, known as ‘deffun’, consisting of lime-cement, crushed earthenware and water. This cover acted as a waterproof layer against the ingress of rainwater [44]. To ensure optimum performance, roof areas were kept small, resulting in the building’s various roofs being at different heights. This kept the amount of fill needed to create falls to a minimum. In addition, it served to keep the thickness of the overlying deffun layer small to avoid cracking due to thermal stresses, which generally peak during July and August, when the intensity of solar radiation may reach circa 8 kWh/m^2^/day on a flat roof [45]. An outline of the roof engineering solutions and construction details of traditional Maltese architecture is given in Mahoney ([42]: 79–80) and Hughes ([40]: 196–197), respectively. These solutions and details are reproduced in Table 6 and Fig. 11.

**Table 6:** Roof-building engineering solutions in traditional architecture in Malta [2, 40, 42]

Span (s)	Roof building engineering solutions
0.7 m < s ≤ 2.00 m	2.0 m is the maximum span of a xriek without failing in tension
2.0 m < s ≤ 2.75 m	The effective span of the space at roof level is reduced to 2.0 m (and thus can be roofed by a xriek tal-qasab) by: either sloping gently the walls or adding corbelling (Maltese: kileb) below the roofing slabs
s > 2.75 m	At ground floor level, masonry arches are used at circa 1.2 m intervals with xorok spanning from one arch to the other; at upper levels: the masonry arches are replaced by timber beams.

LGL was readily available locally and the labour involved in quarrying it was cheap, as was the extraction of Coralline Limestone (CL) and its processing for the production of lime. Both types of limestone were quarried by inserting timber wedges in grooves cut in the stone face; the wedges were then soaked so that the timber expands and cracks the bedrock. Timber, pozzolana and iron had to be imported. The island did not have an adequate supply of woodland, and no mineral deposits suitable for the production of pozzolana or iron.

Limestone dimension stones were often quarried at the building site, which had the added advantage of producing space for a lower level and/or cisterns for rainwater collection. D’Amato [46] claimed that the site of the cistern adjacent to the yard provided the building stones for Casa Ippolito. A closer inspection of a section of the exposed limestone at the cistern and the dimension stones of the house revealed that the limestone is indeed identical. However, this is not incontrovertible proof that it came from this specific location, as no historical or other empirical evidence has been found to support this claim. Using construction stones which originated close to the environment of deposition ensures a more stable environment for the fabric once it forms a component of the structure.

Lime-based mortar was used to level and fill in the spaces between the two leafs in the double walls. Lime was valued for its permeability, flexibility and aesthetic effect [47]. Permeability allows the movement of moisture, especially in porous limestone such as LGL, regulating the humidity of the fabric and limiting the impact of rising damp by allowing the stone to ‘breathe’ [48]. However, this argument is contested by Joseph Falzon, the former Dean of the Faculty of Architecture and Civil Engineering, the forerunner of the Faculty for the Built Environment, of the University of Malta. Falzon argues that rising damp was limited by the capillary pressure in the stone pores. Irrespective of the mortar used, damp was present to about 1 m above ground level. The deformation of the mortar is more likely to be plastic than elastic. The most important aspect in the control of movement was the typical situation with masonry. The components were small, which meant that deformations were minute and easily accommodated. Stone is not hygroscopic and, besides protecting the built fabric, lime complements its natural texture. Traditionally, buildings exposed to rising damp would have the whole or the first 3.0 m of the walls whitewashed ([40]: 196). In addition, the interior walls would be lime washed, using a traditional mixture of lime and water resulting in a white texture, after they had been smoothed down. In the past, plastering was generally absent from local building construction.

Traditionally, LGL powder, known as xaħx, wash was applied to the exterior. Although this typically washed away after a few years, a certain amount was absorbed by the mortar joint, making the wall more uniform in colour. The present state of the external walls of Casa Ippolito might be due to either never having been xaħx-washed or the xaħx wash having been obliterated by rainfall over the centuries. Other treatments would have attracted attention, especially in a landscape close to the sea. However, by the late seventeenth century, when the Casa was erected, the Ottoman Empire had ceased to be a threat in the central Mediterranean ([49]: 135) (an opinion not shared by Hughes ([40]: 4). Concrete with reinforcement and cement-based pointing and plastering dating to the later part of the 20th century were applied to the external walls of the mill and the part of the stairwell which belonged to the later phase of the building, overlooking the yard. Such interventions enabled damp to rise to higher levels, preventing LGL from ‘breathing’, thus resulting in further deterioration of the host fabric.

Selective intra-burrow cementation and preferential erosion of the surrounding poorly cemented sediment account for the observed alveolar weathering [50]. The mineralogy of the burrow infill is both qualitatively and quantitatively different from the host sediment [51]. The unlithified sediment introduced through bio-retexturing modified the permeability and porosity of the original depositional fabric and thus effected the capillary intake of water from the ground, which impinged on its weathering [51, 52]. Severe honeycombing can be observed where moisture penetration is present. The issue of preferential weathering can be seen as the result of natural processes occurring within a secondary geodiversity structure (the ruins). Limestone brought from elsewhere for use in buildings represents a secondary geodiversity product; such stones are carefully selected, quarried and transported for their specific qualities: they have human values and, potentially, functional/system support values [53].

**Fig. 12:**
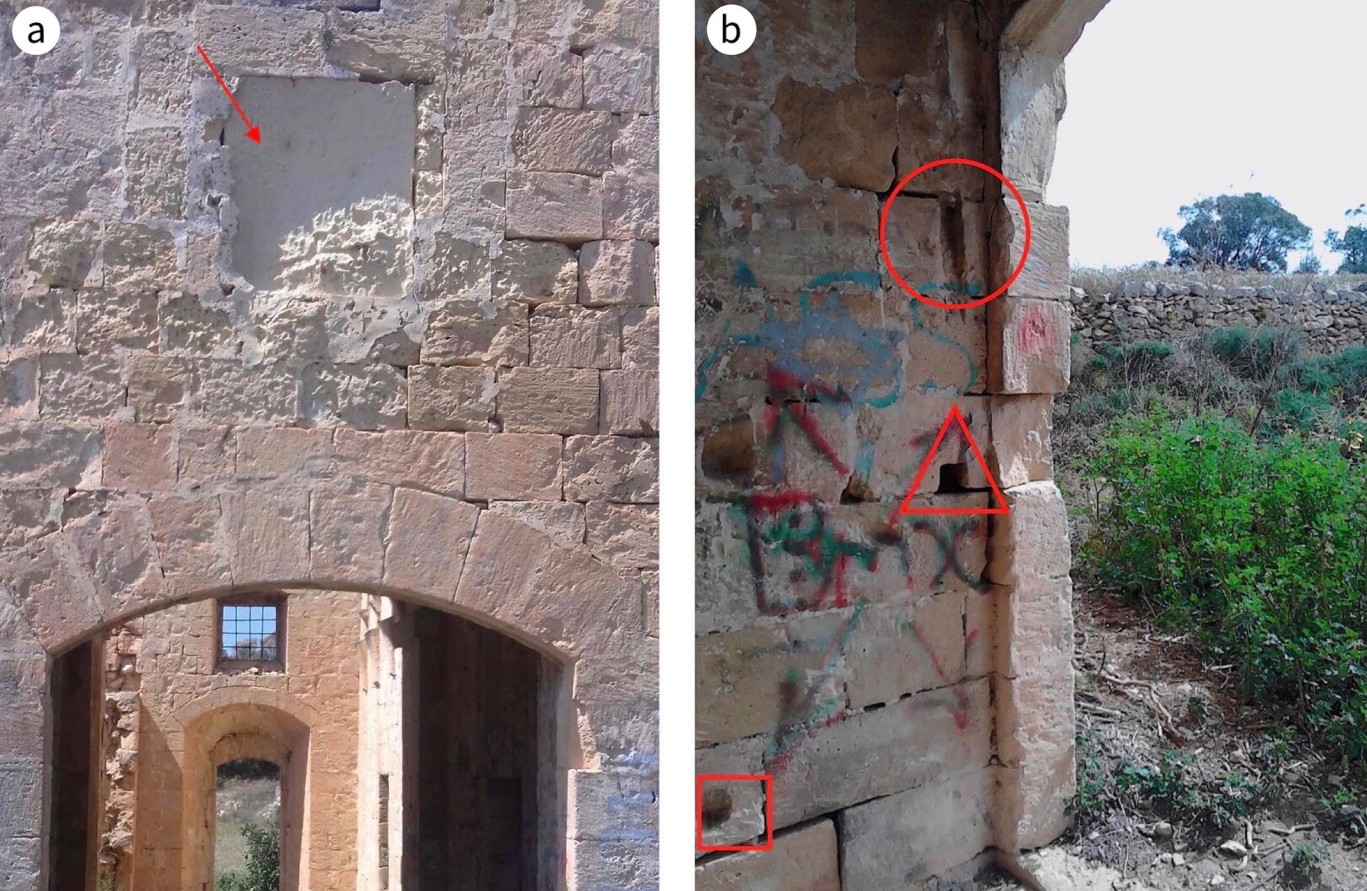
**a** The damage to the inscription (indicated by marker) and the surrounding fabric on the main doorway. **b** The main entrance was bolted at three levels: by means of a bar pivoted on one of the door leafs at the top (circled), a bar in the middle (triangle) and another at the bottom (square)

Freshly quarried LGL must be sculpted within the first four years, after which the stone forms a hard crust. If not used within this period, the stone would either be left uninscribed or replaced. If the hard crust is damaged, the fabric deteriorates, with negative effects on the adjacent limestone ([40]: 199). The damaged inscription on the main doorway—which a century ago was still decipherable despite the stone being heavily deteriorated^8^—and the surrounding fabric exhibit this kind of deterioration (Fig. 12a).

Honeycombing is present, notably at circa 1.3 m above ground level. Differential weathering on the elevation along the public road is evidence that inferior lithostratigraphic beds of LGL were used in the later phase of the building (Fig. 8). This contrasts with the choice of fine LGL and CL ashlar blocks in the early phase of the building, where the exposure was similar. This change in the choice of stone could also imply the involvement of different masons, the former more versed in the craftsmanship of building material than the later one. The dimension stones on the exterior of the elevation dating to the early phase have weathered well, showing the type of weathering that is usually associated with limestone initially naturally treated through quarry sap; once it dries up, the fabric is at its hardest and has maximum weather resilience (Fig. 10a). The state of preservation of this part of the south-east facing elevation further reinforces the view that the LGL used during the later phase was of poorer quality, as it has deteriorated faster than the corresponding LGL used in the early phase (Fig. 4b); stone surfaces exposed to the south generally deteriorate much less than those exposed to the north.

### Geoculture as wellbeing

Ruins can provide insights not only regarding the shelter but also regarding the sensory experience of living in the space. The primary needs of humans, in Abraham Maslow’s hierarchy, are physiological; these include air, drink, food and shelter [54].

Given the rural character of the area and its close proximity to the sea, securing the dwelling against unauthorised entry was a priority. Timber apertures, all opening inwards, were used, enhanced with iron grills. The frames of these apertures, circa 70 mm in thickness, were fixed directly onto the stone rebate. In contrast to the door leading to the open balcony, there is no evidence that the main entrance door had an independent fan light to allow air and light into the hall. Such a detail was probably integrated into the design of the opening; otherwise, unlike the other spaces of the house, which were well lit and ventilated, the hall would have been dark and poorly ventilated. This main door was bolted at three levels:

1. At the top: there are two channels, both 50 mm wide and circa 370 mm long, set at the same height on either side of the door jamb (Fig. 12b). These grooves vary in depth along their length: viewed from the hall, the one on the right is 0 mm at the top and 40 mm at its bottom; the one on the left ranges from 0 mm at the bottom to 80 mm at the top; they appear to mark the outer edges of a circle. This implies they accommodated the clockwise motion of a bar, most likely made of timber, which revolved around a pivot set into the inside of the door. When the bar was in a vertical position the door was unlocked, and when swung clockwise into a horizontal position, its ends would lock into the groves, securing the door. This mode of bolting was rare in Malta, although a similar mechanism can be found on the old entrance door of the Kitchen Garden (adjoining the official residence of the President of Malta, a building dating to the early part of the seventeenth century) ([55]: 180–186). There is no evidence the door was secured from the outside.
2. In the middle: a bar (most likely timber) was manually placed across the door.
3. At the bottom: a horizontal bar (most likely timber) was manually placed from the wall jamb to the middle of the door.

Window openings were secured by iron grills, implying that the windows opened inwards. Only two have survived but there were probably others, as evidenced by the corrosion-related cracks and/or anchoring holes present in the lintels and the jambs.

Rising damp occurs when water is drawn up through the material of the wall by means of capillary action. In a modern building, rising damp indicates either the absence of a damp proofing course (DPC), the bridging of the DPC, or failure of the DPC membrane. Casa Ippolito was erected around two centuries before the Sanitary Laws and Regulations [56] stipulated the mandatory use of DPCs to counteract the dire public health effects of humidity and rising damp. Nevertheless, the masons of the time knew full well about the problem of rising damp, and applied the technologies of the day to avoid it, choosing the more compact CL to construct the walls of the lower level underlying the corridor, thus providing natural damp proofing to the ground floor. The use of this fine hewed stone would have been a deliberate decision: it was harder to quarry and work into blocks than LGL. Some were not from MM and must have been imported to the site from other parts of the island. It is possible that the builders were recycling these stones, but even then, the decision to use them would not have been taken casually; to carry and handle such dense limestone was non-trivial. The 17th-century builders would have introduced this kind of limestone not because they were compelled by law but because they deemed it to be good building practice. Another method of limiting rising damp was through the introduction of a ventilated basement below the building, but this was not applied in the case of Casa Ippolito. Ventilated basements were typical in urban and rural tenements erected on LGL formation, such as the buildings in Valletta. Other cases of rising damp found in parts of the external walls may be due to bridging; given the capillary absorption of LGL [24], the ingress of water with soluble salts from the ground deteriorated the dimension stones immediately above ground level. The rule of thumb among local builders is that dampness rises to ~ 1 m above the source causing it (Salvatore Bondin, personal communication).

One popular method of climatic modification, not used in Casa Ippolito, was the construction of a loggia running along the length of the south-facing facades which provided a buffer zone to the elevations exposed to direct sunlight and protection from rain. This architectural element was once used extensively in the Mediterranean region, notably in Italy, Greece and Spain. Direct solar radiation could not penetrate the interior because the roof of the loggia served as a canopy. These considerations affected the physical and psychological wellbeing of the users: warm, dry walls and the absence of dampness are important elements of a healthy building.

Geological materials in heritage masonry buildings might be freshly extracted (as with dimension stones), produced (in the case of lime) or recycled. At Casa Ippolito, no construction waste was evident on site. All the quarried stone was used, either as building elements or as components in a mix. Double-leaf ashlar masonry provided thermal mass and thus improved indoor climatic conditions; the thicker the wall, the higher the insulation value. The stone mass absorbed heat slowly, releasing it gradually during the colder season. This had the effect of reducing the extremes of temperature and causing a time-lag between changes to the external and internal conditions. The more massive a building, the cooler it would be in summer and the warmer in winter because indoor temperature fluctuations are reduced and the time lag increases. Eventually, if the building is massive enough, such as in an earth building, then the indoor temperature stabilises at the average temperature of the locality. The mass of masonry construction is a dynamic thermal insulant due to the properties of the geological materials used—stone, soil and lime-based mortar. The thickness of internal walls has no bearing on thermal insulation but does contribute to thermal mass; they adjust to the ambient temperature.

Casa Ippolito was a self-sufficient, sustainable household in the sense that it harvested water and produced food from agrarian land forming part of the property, in a manner typical of the times. Historically, country residences were self-sustainable independent units for human survival grounded in zero-waste generation. Water, a primary need for survival, was recycled. Rain water was collected for potable use in cisterns while waste water (grey and brown) was used to irrigate crops.

Until the early twentieth century, agrarian land was valued higher than built-up land. Fields were a resource which secured a person’s living; in contrast, the value of developed land was negligible. Reducing the thickness of the walls on the second storey, and consequently gaining more floor space, was not thought about in terms of the fiscal value of built-up land, as such land was cheap. What mattered was the cost of building: the less stone used, the cheaper it was to erect the building.

### Architectural ruins as secondary geotouristic product

With three World Heritage Sites, first designated in 1980, Malta is second only to the Holy See in terms of heritage density [57]. Protection of heritage has been a priority for successive governments, although public policies geared towards investment in the historic and architectural environment are often met with resistance from business interests. National authorities such as the Planning Authority, the Superintendence of Cultural Heritage and Heritage Malta (a national agency for museums, conservation practice and cultural heritage), along with various NGOs, all work to safeguard the country’s heritage [12].

Returning to the discussion of geodiversity, it could be argued that the act of conserving the ruins of anthropogenic structures constructed from local geological materials is a strategy to protect local geodiversity, making it an act of ‘geoconservation’, defined as “the act of identifying and protecting valuable elements of the abiotic environment. The variability of elements within the abiotic environment can be assessed and described… using the term ‘geodiversity’” ([58]: 2). Not all of an area’s geodiversity has value that justifies the implementation of a geoconservation strategy [59]. However, in the case of architectural ruins such as Casa Ippolito, their geocultural value is not limited to the local provenance of the building materials but encompasses the material culture in its totality, both tangible and intangible—that is, both building construction and the skill of the artisans who created it.

State-owned heritage sites are managed by a single national entity, Heritage Malta, while those in private hands are owned by individuals with differing values and expectations, making the heritage management challenges greater. Privately owned sites (such as Casa Ippolito) may fall into ruin for a number of reasons, ranging from litigation (such as inheritance disputes) to neglect; the site might also be turned over to new development. The tight-knit nature of Maltese society, in a small island state where most original inhabitants are interrelated through consanguinity and familial affiliations, means that historical disputes, divergent agendas and various other undercurrents inevitably play a role in matters such as heritage protection. Local individuals have differing perspectives on cultural heritage—a building which, to one viewer, merits heritage protection might represent a speculative development opportunity to the owner.

As a manmade/anthropogenic structure erected in local limestone, Casa Ippolito fits into the category of secondary geodiversity. Geodiversity represents an important resource for tourist and recreation activities, with strong geoeducation and geotourism potential [60]. Architectural ruins can satisfy niche interests, for example, in the anthropogenic use of geological material. To give an example from Casa Ippolito, the cistern is a recycled mining landform, where a pit left from quarrying has been repurposed for storing water. If visiting, learning from and appreciating sites of geological interest falls within the remit of primary geotourism [61], it could be argued that visiting architectural ruins is a form of secondary or anthropogenic geotourism. However, before such visits can take place, all necessary restoration works must be undertaken to ensure the site meets modern standards for health and safety and accessibility. Furthermore, if not protected from rainwater and vandalism, ruins can suffer considerable damage, so a covered shelter may be required to protect vulnerable parts of the structure. Even if such interventions are carried out, given the high density of important heritage sites in Malta, such ruins remain of relatively minor importance and will not therefore play a major role in the current cultural tourism market. Nevertheless, there are other niches which are either under-valued or not yet being exploited, including study tours and educational fieldwork trips for students and scholars interested in anthropogenic and geocultural research; in other words, the site has substantial secondary geoeducational potential.

## Conclusions

An exemplar of a 17th-century aristocratic residences, Casa Ippolito was left derelict for over a century before it was awarded the necessary degree of heritage protection. Its architectural features make it a valuable primary source for the history of the art and science of building in Malta. The knowledgeable and creative use of locally sourced materials, as well as the architectural statement expressed through its simple yet elegant construction, make Casa Ippolito a classic example of Malta’s secondary or anthropogenic geodiversity. It is a declaration, written in stone, of the geological and architectural heritage of Malta.

The history of building engineering and construction in the Mediterranean is a source for contemporary, contextual, architectural design solutions for the region [62]. Casa Ippolito is a typical architectural ruin, an illustration of anthropogenic geodiversity which constitutes an essay in building engineering and construction techniques and in the materials available at the time.

Architectural ruins are primary sources for comprehending the local built heritage; they represent a laboratory of building physics. The elements of architecture can be seen as a vocabulary expressed in masonry. As this case study has illustrated, the researcher can access this physical ‘essay’ through both on-site evidence from the ruins and their surroundings, and through examination of the historical documentation, in order to produce an accurate reconstruction of the building in its original geophysical context, such as the bedrock on which it was erected and how the site selection may have been dictated by the accessibility of building materials. Ruins offer insight into the anatomy of a building—its structure, layout and aesthetics—as well as its dynamics, going beyond the mere building materials. For example, the researcher can observe how local climatic conditions, the orientation of a building on site and the position of apertures affected the influx of daylight, passive solar heating and cooling, and natural ventilation—all physical factors which had a bearing on the users.

Ruins can act as sites for cultural tourism, but they are also, whether for researchers or for thoughtful visitors, a dissection through the essence of architecture. Architectural history enables the reconstruction of not only spaces from ruins but places for users. Architectural ruins offer insight into the dynamic between the local geoculture and the wellbeing of the humans that once used the building. Heritage buildings such as Casa Ippolito were sustainable, environment-friendly units which brought together the natural properties of materials and cunning human artifice to optimise light, thermal insulation and ventilation to create a pleasant liveable space.

Understanding the dynamics and construction techniques of the past can provide useful insights into how to design or upgrade modern buildings to be more sustainable and environmentally sound. While a building lies in ruins, the construction methods and approaches to building dynamics illustrated in its remains provide invaluable lessons for sustainable architecture today. To use a statement attributed to Gustav Mahler, “tradition is tending the flame, it’s not worshipping the ashes” ([63]: 104).

## Data Availability

All data generated or analysed during this study are included in this paper.

## Footnotes

1 NMA: note on roots of title to ownership of Casa Ippolito, p. 1 (in Italian).

2 NMA: Council of Europe Monument Number.

3 NMA: note on roots of title to ownership of Casa Ippolito, p. 1 (in Italian).

4 NMA: note on roots of title to ownership of Casa Ippolito, p. 1 (in Italian).

5 NMA: Council of Europe Monument Number continued.

6 NMA: extract from the Deed of Notary Pietro Mifsud, dated 7th December,1893 (in Italian, translated by the author).

7 NMA: extract from the Deeds of Notary Pietro Mifsud 7th December, 1893.

8 NMA: note on roots of title to ownership of Casa Ippolito, p. 1 (in Italian).
